# Comprehensive Research of FSW Joints of AZ91 Magnesium Alloy

**DOI:** 10.3390/ma16113953

**Published:** 2023-05-25

**Authors:** Krzysztof Mroczka, Stanisław Dymek, Aleksandra Węglowska, Carter Hamilton, Mateusz Kopyściański, Adam Pietras, Paweł Kurtyka

**Affiliations:** 1 Department of Materials Engineering, Faculty of Materials Engineering and Physics, Cracow University of Technology, 31-155 Kraków, Poland; 2 Department of Surface Engineering and Materials Characterization, AGH University of Science and Technology in Krakow, 30-059 Kraków, Poland; gmdymek@cyfronet.pl (S.D.); mateusz.kopyscianski@gmail.com (M.K.); 3 Welding Centre, Łukasiewicz Research Network—Upper Silesian Institute of Technology, 44-100 Gliwice, Poland; aleksandra.weglowska@git.lukasiewicz.gov.pl (A.W.); adam.pietras@git.lukasiewicz.gov.pl (A.P.); 4 Department of Mechanical and Manufacturing Engineering, Miami University, Oxford, OH 45056, USA; hamiltbc@miamioh.edu; 5 Innerco sp. z o.o., Materials Research Laboratory (MRL), Jadwigi Majowny 43A St., 30-298 Kraków, Poland; pkurtyka@icloud.com; 6 Materialica+ Research Group, Poland

**Keywords:** FSW, AZ91 magnesium alloy, micromechanical tensile test, numerical model

## Abstract

For the friction stir welding (FSW) of AZ91 magnesium alloy, low tool rotational speeds and increased tool linear speeds (ratio 3.2) along with a larger diameter shoulder and pin are utilized. The research focused on the influence of welding forces and the characterization of the welds by light microscopy, scanning electron microscopy with an electron backscatter diffraction system (SEM-EBSD), hardness distribution across the joint cross-section, joint tensile strength, and SEM examination of fractured specimens after tensile tests. The micromechanical static tensile tests performed are unique and reveal the material strength distribution within the joint. A numerical model of the temperature distribution and material flow during joining is also presented. The work demonstrates that a good-quality joint can be obtained. A fine microstructure is formed at the weld face, containing larger precipitates of the intermetallic phase, while the weld nugget comprises larger grains. The numerical simulation correlates well with experimental measurements. On the advancing side, the hardness (approx. 60 HV0.1) and strength (approx. 150 MPa) of the weld are lower, which is also related to the lower plasticity of this region of the joint. The strength (approx. 300 MPa) in some micro-areas is significantly higher than that of the overall joint (204 MPa). This is primarily attributable to the macroscopic sample also containing material in the as-cast state, i.e., unwrought. The microprobe therefore includes less potential crack nucleation mechanisms, such as microsegregations and microshrinkage.

## 1. Introduction

The first indications of the probable occurrence of magnesium (Mg) as an element in chemical compounds date back to around 1640 [1]. At that time, bitter springs were already known in the vicinity of the English city of Epsom. Later research indicates that the taste and properties of Epsom’s water and salt may have been due to the presence of Mg [1]. Magnesium was discovered in minerals by A. Maggraf (1759) and in organic compounds by A.F. Fourcroy and N. Vauquelin (1803). The receipt of Mg as a silvery metal is attributed to A Bussy in 1830 [1].

Mg occurs in the Earth’s crust at about 2.5%. The use of Mg to create construction materials results from the possibility of its strengthening and forming. At the same time, Mg has a very low density, i.e., about 33.6% lighter than Al and Ti, and 75% lighter than steel [2]. Mg is strengthened by additives [3,4,5], heat, and plastic treatment [6], e.g., ECAP technology [7,8]. Numerous alloys are formed based on a Mg matrix, and the most frequently tested magnesium alloys include AZ31, ZE41, AZ61, AM60, AZ61, AZ80, and AZ91 [9,10,11,12,13,14,15,16,17]. The most common methods used to produce elements from Mg alloys are cast, die-cast [18], cast-rolling [19], rolling [20], squeeze cast [21], and extrusion [10,22,23]. Brittleness is the main problem in the processing and use of Mg and its alloys. This case has been extensively described in [24], where methods of producing Mg-based materials with simultaneous improvement of plasticity and strength have also been indicated.

One of the more interesting Mg alloys is the AZ91 alloy. The physical and mechanical properties of the alloy are given in Table 1 and Table 2, and the chemical composition is given in Table 3.

AZ91 is uniquely used, and casting elements, composites [27,28], and extruded elements [29] are produced from this alloy. It is also being considered and studied for the possibility of hydrogen accumulation [30]. Like other Mg alloys, AZ91 is very reactive to the environment. For this reason, a surface remelting treatment is used [31], or layers are developed on the surface to improve corrosion resistance [32]. Magnesium alloys are commonly utilized in the aviation, automotive, and medical industries, as well as many others [33,34]. Joining elements made of Mg alloys is typically achieved with laser welding [35,36,37] and other fusion welding methods (e.g., TIG [38]).

One of the most effective methods of joining Mg alloys is friction stir welding (FSW) [39]. FSW was developed at TWI (The Welding Institute, Cambridge, UK) in 1991 [40]. The basis of this method is plastic deformation (stirring) of the joined materials by a specially profiled FSW tool. The process is carried out in the solid state without shielding gases, and the heat input to the workpieces is significantly less than that for conventional welding methods. In order to obtain sufficient plasticity of the material (especially for Mg alloys), it is necessary to control the temperature of the process. Since Mg-matrix materials have a hexagonal crystallographic lattice, the number of slip systems at room temperature is small, thus presenting some challenges in the plastic deformation of these materials [20,41,42]. Temperature and material flow control in FSW is carried out by selecting the shape of the FSW tool and welding parameters. Numerical models are developed to enhance the understanding of the processes occurring during FSW and to predict its effects [40,43,44,45]. Characterization of FSW joints most often includes the following: macro- and microstructure, hardness distributions across cross-sections, and tensile strength tested on samples cut perpendicular to the weld direction. In addition to these tests, this study also includes the results from new tests that enable the determination of the strength from specific areas of the joint—micromechanical tests. It should be noted that the term *micromechanical* testing is used in the literature with various meanings, e.g., to describe micro-beam bending tests [46], to characterize smooth and rough surfaces [47], or to investigate interlaminar crack propagation [48]. In this work, however, the performance of tensile tests on small-scale samples excised from individual areas of the joint is described. This method allows the examination of specific areas from larger samples or when the test material is small. The test results are analogous to those for large-scale tests; i.e., the stress–strain curve is obtained [49,50]. In addition, the results presented in this work refer to joints obtained with a relatively low rotational speed of the FSW tool, whereas such tests are typically performed on joints manufactured with higher rotational speeds, 710–2000 rpm. Additionally, most published works dedicated to the FSW welding of Mg alloys focus on the AZ31 Mg alloy, rather than AZ91 [39].

In summary, this work contains several new issues not yet described in the literature on the subject. The most important are the use of low rotational speeds and relatively high linear speeds of the FSW tool for welding the AZ91 magnesium alloy, the application of micromechanical tests to determine the mechanical properties of the weld, and the preparation of a numerical model of temperature distribution for the welding parameters used.

## 2. Materials and Methods

The magnesium alloy AZ91 (Table 3) was friction stir welded with the parameters given in Table 4. The FSW tool used in the experiments consists of a shoulder of 24 mm diameter with a spiral groove on its working surface and an 8 mm diameter, threaded cylindrical pin. The tool is made of high-speed steel 1.3343 (SW7M, HS6-5-2C). The macro- and microstructure were investigated by light microscopy following surface etching from the following reagents: C_6_H_2_(NO_2_)_3_OH, 4.2 g; CH_3_COOH, 1 cm^3^; H_2_O, 10 cm^3^; C_2_H_5_OH 96%, 75 cm^3^. Scanning electron microscopy, including energy-dispersive X-ray spectroscopy (EDS) and electron backscatter diffraction (EBSD), was used to characterize the microstructure and fracture specimens.

Concerning the metallographic preparation of specimens for microscopic examination, it should be noted that magnesium and its alloys are difficult materials to prepare. The high reactivity of magnesium with water precludes the use of preparations containing water. This material is also quite susceptible to sandpaper particles being lodged into the surface during the first stages of grinding the surface of the samples. In many cases, this is also the reason for the occurrence of black spots on microscopic images. These are places where particles were introduced during preparation. The procedure used to prepare the surface for testing consisted of four stages: grinding on #360 abrasive foil, and then polishing on a pad using 9 μm, 3 μm, and 1 μm diamond suspensions. For SEM-EBSD studies, the deformed layer was removed from the surface using an ion thinner, directing the ion beam at a minimal angle.

Chemical analysis using an SEM-EDS system was carried out on fractures of samples broken in mechanical tests. The analysis parameters (accelerating voltage and spot size) were selected for the elements present in the AZ91 alloy and according to the detector manufacturer’s instructions. The analysis was carried out pointwise with automatic recognition of elements. The elements that did not occur in the alloy and came from contamination of fractures were removed from the analysis.

**Figure 1:**
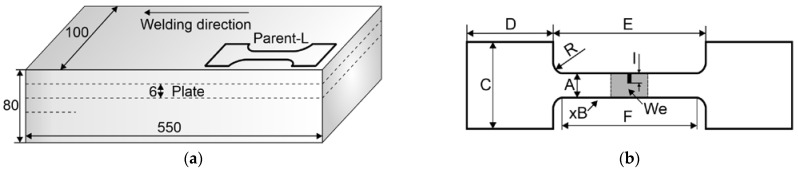
Material and specimen preparation: (**a**) cutting out of plates from cast block of AZ91 alloy; (**b**) tensile test specimens and the location (We) of the weld and notch (I) in Macro-V specimen.

**Figure 2:**
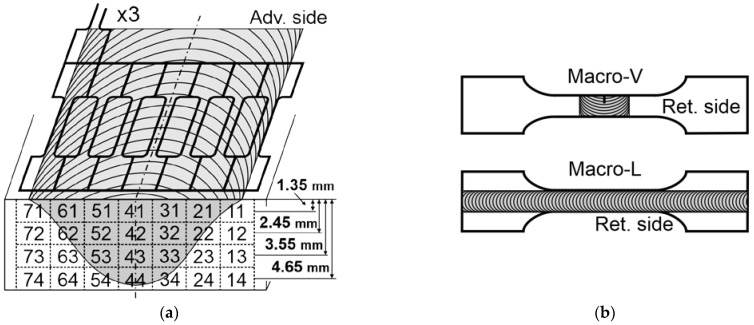
Tensile test specimens: (**a**) places of excise of the Micro specimens, three for each section from No. 11 to 74; (**b**) cutting method for Macro-V and Macro-L specimens; Ret.—retreating side.

For mechanical properties, tensile tests and microhardness tests were performed. Three types of tensile test specimens were used: Micro—local property testing; Macro-V—specimen with the weld, oriented perpendicular to the welding direction and weld area, containing a notch (I) 3 mm long and 0.3 mm wide (We in Figure 1b); Macro-L—longitudinally oriented to the weld direction. Table 5 presents the dimensions of the tensile test specimens concerning Figure 1. Excise locations for the Micro specimens concerning the welds’ macrostructure are shown in Figure 2a. Wire electrical discharge machining (WEDM) was used for the cut-out.

## 3. Results

Detailed tests of the microstructure, mechanical properties, and weld welding conditions (forces and torque) were carried out in relation to the following variant: rotational speed 355 rpm, linear speed 112 mm/min. The second variant of welding at the same low rotational speed of the pin but with a slightly higher linear speed (Table 4) also achieved a weld without external or internal defects. However, precipitation bands on the retreating side occurring at the weld face are more extensive under these conditions than those for which the linear speed is 112 mm/min. Therefore, welds made at the lower linear speed were selected for detailed studies and are presented below.

### 3.1. Forces in Welding

**Figure 3:**
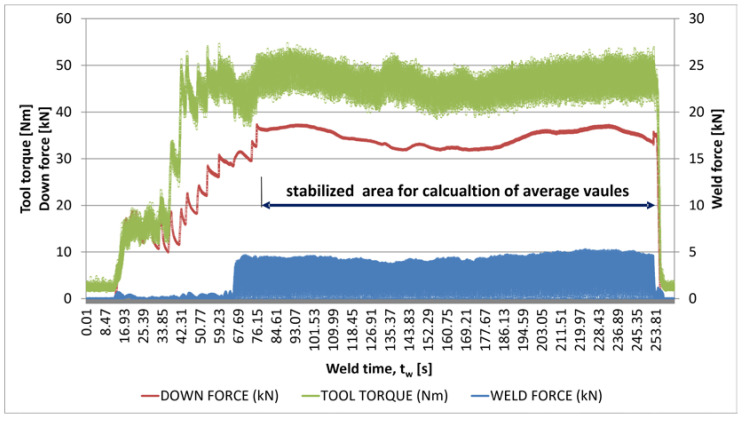
Forces and tool torque of welding.

In the first stage of the research, the parameters of the welding process were determined. The tests were carried out with a specialized machine head that enabled the measurement of forces, torques, and temperature of the FSW tool. Figure 3 presents graphs showing the forces and tool torque for the entire joint fabrication process. Based on these data, the average values given in Table 6 were calculated for the stable range (marked in Figure 3). The first 55 s of the diagram highlights the period during which the tool was inserted into the material and held. The significant fluctuation in the weld parameters during this dwell time is due to manual control of the tool movement. The linear motion of the tool (welding) then started after approx. 67 s, i.e., immediately after the tool reached the horizontal target position in the material. Stabilization of the parameters took place after approx. 13 s from the start of the linear motion, which corresponds to the tool travel of approx. 24 mm. Taking into account the final welding phase, it is assumed that the stable range of the process lasted approx. 174 s, which corresponds to approx. 324 mm of the weld.

The graph (Figure 3) shows a decrease in parameters after approx. 59–104 s from the start of the linear motion of the tool (135–180 s of total time), which corresponds to a joint length of approx. 110–194 mm. This decrease may be due to the geometry of the welded plates. It must be remembered that the plates were mechanically cut from a cast block. They were then milled to a thickness of 6 mm. During these mechanical treatments, the internal stresses existing in the casting are released. In addition, the material was cut and milled without using coolant due to the reactivity of the coolant with magnesium. They were carried out at very low speeds, but regardless of this, there were local increases in temperature, causing additional stresses. In addition, magnesium is a difficult material to machine due to the significant elastic deformations accompanying this process, and each cast material exhibits heterogeneity. All these factors may cause minor differences in the thickness of the prepared elements and their distortions, e.g., twisting along the length. During welding, the plates were clamped on the welding table, and this could also introduce stresses which, together with the change in material temperature during the process, may affect the process parameters.

### 3.2. Macro- and Microstructure

Macrostructure studies are an important part of the analysis of joints, as they enable the assessment of the size and shape of individual features, i.e., the thermomechanically affected zone and the weld nugget. The depth of the process zone on the cross-section related to the pin can also be identified. However, a critical characteristic to examine is the presence (or absence) of defects such as cracks, voids, segregations, and S or zig-zag lines [51]. 

**Figure 4:**
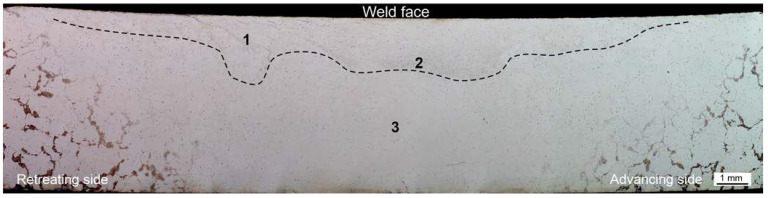
Macrostructure of weld fabricated 355 rpm/112 mm/min; 1, 2, 3—places presented in Figure 5.

**Figure 5:**
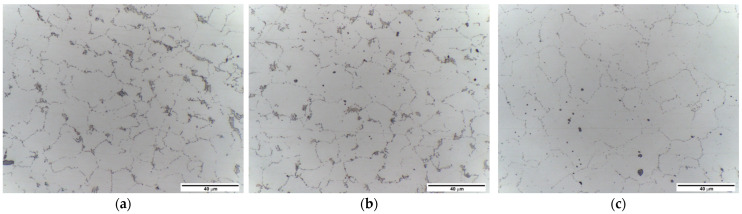
Microstructure of weld fabricated 355 rpm/112 mm/min, relative to places in Figure 4: (**a**) 1; (**b**) 2; (**c**) 3.

Figure 4 shows the investigated joint macrostructure. Tests revealed no defects in the joint in the form of voids, cracks, discontinuities, porosity, or segregation. However, unique microstructural features were discerned near the weld face as compared to the nugget and areas further away from the weld face. The microstructure of the weld nugget is shown in Figure 5c. It is the area marked with the number 3 in Figure 4. The nugget consists of grains with precipitates located on the boundaries, which distinctly highlights the grain size and morphology. The microstructure in the zone at the weld face is shown in Figure 5a,b. In Figure 4, the tested places are marked with numbers 1 and 2, and the border of the zone is denoted with a dashed line. The microstructure in this area of the joint consists of grains, grain boundary precipitates, and clusters of precipitates. Clusters often occur in places where several grains connect. The largest number of such clusters, also forming bands, occurs on the retreating side.

### 3.3. SEM-EBSD

**Figure 6:**
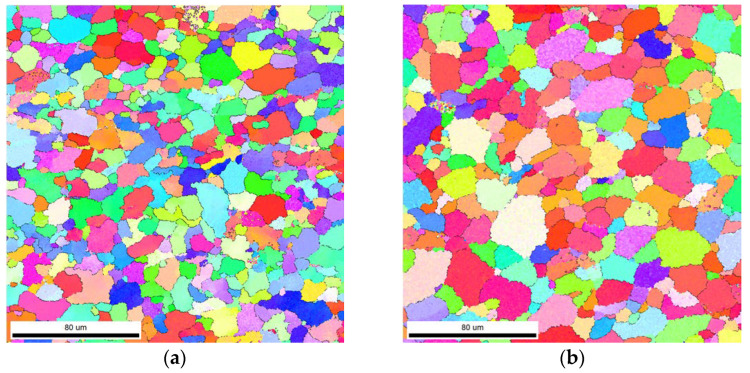
SEM-EBSD microstructure: (**a**) place close to weld face; (**b**) weld nugget. The colors are assigned to the crystallographic orientations of grains.

**Figure 7:**
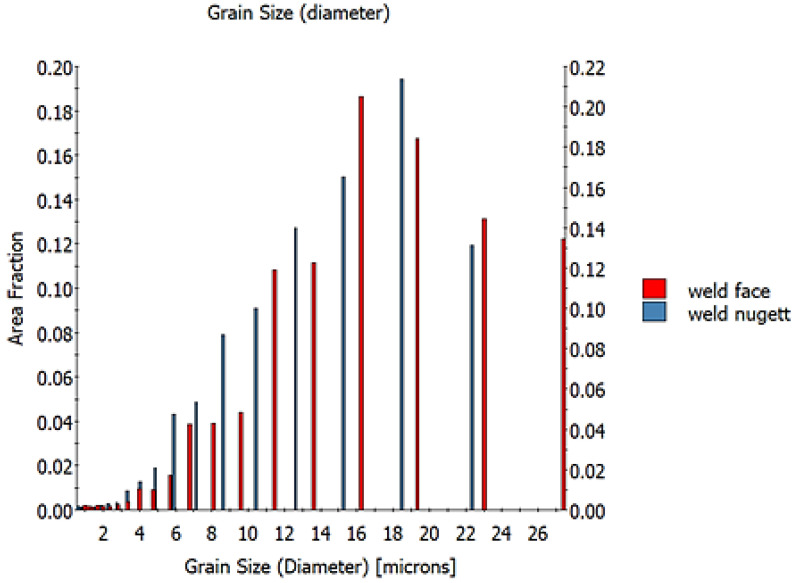
Grain size histogram based on SEM-EBSD analysis (Figure 6).

Scanning electron microscopy (SEM) with an electron backscatter diffraction (EBSD) detector enables microstructural studies based on recognizing the crystallographic lattice and its orientation relative to a specific reference system. On this basis, the microscope software extracts grains and recognizes the orientation of their crystallographic lattice. After a color is assigned to a given orientation range, grain maps are created. Figure 6 shows the areas at the weld face and the area of the weld nugget. When the data are compared, it can be seen that the grains in the weld nugget are larger than the grains at the weld face. There is also a difference in grain orientation. At the face of the weld, the variation in orientation is greater. Such a microstructure is formed under significant plastic deformation which induces strong dynamic grain nucleation. In turn, the area of the weld nugget is characterized by both larger maximum grain sizes and a wider distribution in sizes. On the basis of EBSD research, it was found that the average grain size is 16.5 μm in the weld nugget and 13.5 μm close to the weld face. The smaller grain size is due to the greater plastic deformation at the top weld. During the process, the shoulder induces significant plastic deformation and dynamic recrystallization, resulting in a smaller grain at the weld face. The grain size histogram for the studied areas is shown in Figure 7.

### 3.4. Hardness

**Figure 8:**
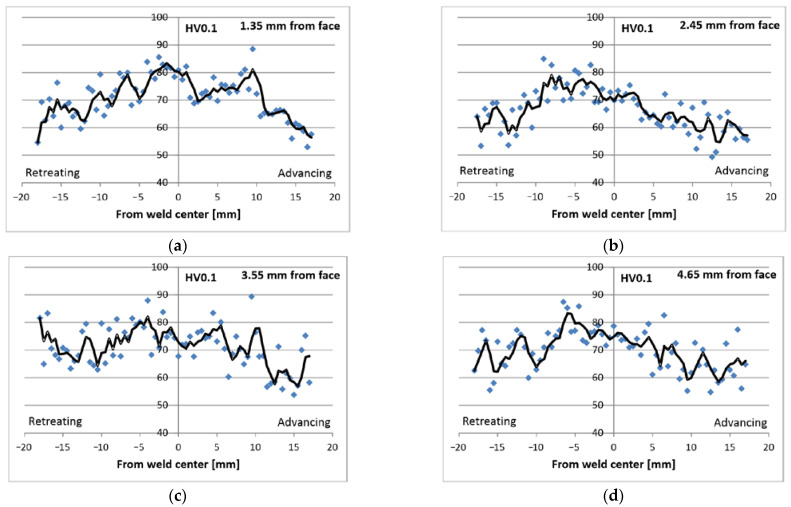
Hardness distribution on cross-section from weld face: (**a**) 1.35 mm; (**b**) 2.45 mm; (**c**) 3.55 mm; (**d**) 4.65 mm.

Figure 8 shows the results of hardness measurements carried out on a cross-section at distances of 1.35 mm, 2.45 mm, 3.55 mm, and 4.65 mm from the weld face. In all cases, the thermomechanically affected zone (TMAZ) area shows a higher hardness than the surrounding areas. In addition, the hardness on the retreating side is higher than that on the advancing side. Regardless of the distance from the face, the highest hardness is approx. 80 HV0.1 in the middle part of the weld or slightly on the retreating side. The lowest hardness reaches approx. 60 HV0.1 and occurs in areas far away from the weld axis (15–20 mm), mainly on the advancing side. Relative to the smallest hardness, the increase is 30%. The graphs show a trend line for groups of results that show clear deviation, especially for data on the lower layers (3.55 mm and 4.65 mm). In these areas, there is a narrower range of material plastically deformed by the FSW tool. The second reason for the deviation of the results is related to the measurement method and the properties of the macrostructure in the micro-areas. The tests used a sampling distance of 0.5 mm between the measurement points and a very small load (100 g). Therefore, the indentations were also small, and their size is comparable to the size of a few grains in the material. It should be noted that the grains show anisotropic properties in terms of the relationship between the hardness and crystallographic orientation of the grain. The particularly large spread in the results in the lower parts of the joint and further away from the center is related to the microstructure after the casting process. These areas were not plastically deformed, so no recrystallization occurred; rather, there is a heterogeneous dendritic microstructure.

### 3.5. Tensile Test Results

Strength tests were carried out in two variants: tests of specimens of standard dimensions and micromechanical tests, i.e., on small-scale specimens taken from individual places of the joint. The tests were carried out on the samples described in Figure 1 and Figure 2 and Table 5.

#### 3.5.1. Macro Specimens

Tests on conventional specimens permitted the determination of the strength of the parent material and the overall joint. These test results are given in Table 7. The parent material was tested in two directions, i.e., parallel to the welding direction (Parent-L) and perpendicular to the welding direction (Macro-V) (Figure 1a). The difference in the obtained tensile strength is significant due to the cast microstructure. The direction parallel to the welding direction (the longer edge of the plate (Figure 1a)) is also the direction perpendicular to the growth of dendrites in the casting. In this orientation, tensile forces act on the dendrite boundaries and interdendritic channels, i.e., weak points of the microstructure. In contrast, the orientation perpendicular to the direction of welding (Parent-V) is parallel to the growth of dendrites. In this case, the tensile forces primarily act directly on the dendrites along their main growth axis. The dendrite is a matrix of Mg with alloy additions in the amount resulting from non-equilibrium solidification with a stable concentration gradient before the crystallization front. For the welds, loading the specimens cut perpendicularly to the weld direction (Figure 2b, Macro-V) without notching showed higher joint strength each time. The specimen broke in the region of the parent material. Since plastic deformation and recrystallization occur in the weld region, these areas of joints are strengthened relative to the parent material and its as-cast microstructure. Moreover, the parent material is the weakest in this load direction, as previously mentioned. However, in order to promote fracture in the joint, a notch was made on the specimen (Figure 2b, Table 5). The test results showed that the joint is stronger in this direction (Macro-V) than in the longitudinal direction (Macro-L, Figure 2b, Table 5).

#### 3.5.2. Micro Specimens

**Figure 9:**
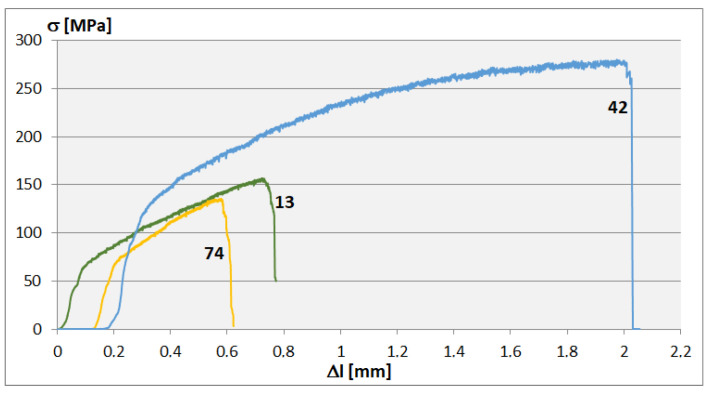
Curves of tensile tests—Micro specimens; exempla for locations 13, 42, and 74 relative to Figure 2a and Table 8 and Table 9.

Tensile micromechanical testing is a novel approach to testing FSW joints. The very small dimensions of these specimens made it possible to study the mechanical properties of specific weld areas. The tests were carried out systematically across the entire joint, dividing the joint into columns and rows. The centers of the rows were spaced from the weld face, similar to the hardness measurement lines (Figure 8). The sampling locations are shown in Figure 2a. Five specimens were cut for each field, and three of them were tested. The results (average values) are presented in Table 8 and Table 9. The highest tensile strength Rm of approx. 300 MPa (Table 8) was recorded for the areas on the retreating side in the middle of the joint. The lowest strength of approx. 130–150 MPa was found in places far from the weld’s axis. Deviations from the average value are minor, mainly for wrought areas. On the other hand, large deviations most often appear in places with a cast structure. Similar observations apply to the yield strength Rp0.2 presented in Table 9. Calculations of this parameter were performed graphically on individual stress/strain curves. Example curves for single measurements from locations 13, 42, and 74 in the weld are shown in Figure 9. These places are also marked in Table 8 and Table 9. When examining the mechanical properties in this way, special attention should be paid to the correct cutting of the samples. Cutting (WEDM) should not be interrupted in the measurement area of the specimen.

### 3.6. SEM Fracture

Breaking the joint in static tensile tests reveals many of the joint’s features. Typically, it allows the assessment of the ductility or brittleness of the material. However, in relation to the entire joint, it is possible to recognize which regions exhibit the features mentioned above, and, more importantly, it is possible to discern adhesion from the continuity of the material produced by metallic bonds on the fracture surfaces. Such places cannot be identified on cross-sections of joints in the microscopic examination, except when precipitates (segregation) are on these surfaces. Adhesion surfaces are the weak points of the joint, and at the same time, they are difficult to detect other than by performing a static tensile test and SEM study of fractures.

#### 3.6.1. Fracture after Tensile Test—Longitudinal Stretching

**Figure 10:**
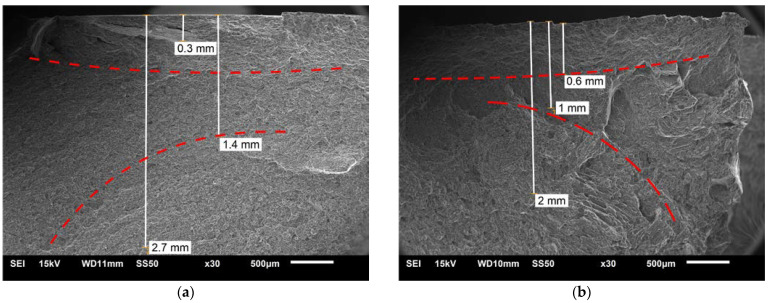
SEM fracture, Macro-L specimen (Figure 2b): (**a**) weld center from retreating side; (**b**) advancing side.

Figure 10 shows the SEM image of the joint fracture stretched along the weld (Macro-L sample, Figure 2b). Individual joint areas can be distinguished at the fracture, characterized by a different structure or directional arrangement of the microstructure. The boundaries of the area near the weld face and weld nugget are marked with dashed lines. In the middle part of the weld from the retreating side (Figure 10a), at an approximate distance of 0.15–0.3 mm from the weld face, there are short delaminations. In contrast, on the advancing side, the breakthrough is developed and has the characteristics of a fragile fracture on the macro scale. It means that this part of the joint is less strong.

#### 3.6.2. Fracture after Tensile Test—Vertical Stretching

**Figure 11:**
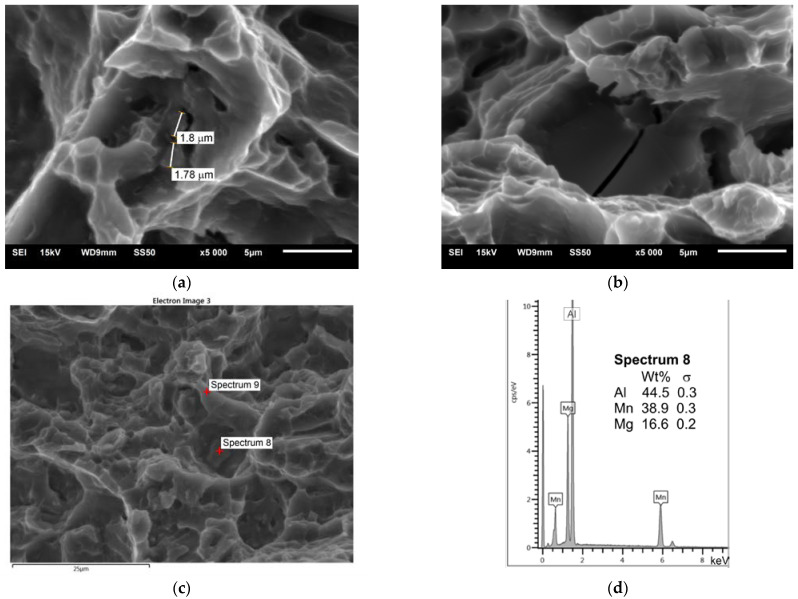
SEM fracture, Macro-V specimen (Figure 2b), 1 mm from weld face, center: (**a**) size of particles; (**b**) brittleness of larger particles; (**c**) EDS analysis of fracture and points; (**d**) EDS spectrum for Spectrum 8—a particle.

Figure 11 shows the SEM microstructure of the fracture of the specimen with a notch (Figure 2b, Macro-V) stretched perpendicular to the direction of welding; it is the area at a distance of 1 mm from the weld face. The fracture in this area is ductile but has limited material flow (plasticity). Two types of particles were identified in this area—small particles, approx. 2 µm, containing Al and Mn (Figure 11a,c,d), and relatively large particles, approx. 10 µm, containing Al. It should be noted that in both cases, the detector also found Mg. 

#### 3.6.3. Fracture after Tensile Test—Micro Specimens

**Figure 12:**
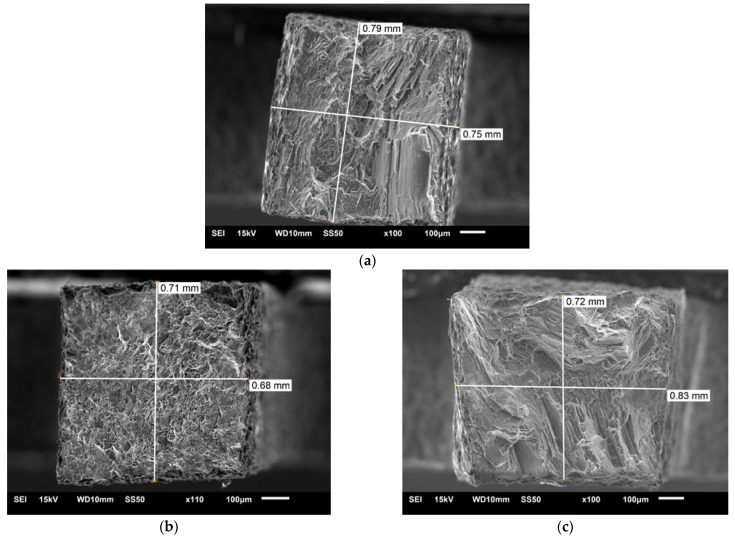
SEM fracture, Micro specimens relative to Figure 2a and Figure 9 and Table 8 and Table 9: (**a**) No. 74; (**b**) No. 42; (**c**) No. 13.

The SEM photos in Figure 12 show fractures of specimens from micromechanical tests for weld areas numbered 74, 42, and 13 (Figure 2a). The fractures of samples 74 and 13 show brittleness, while sample 42 has a fine fracture with ductile elements. The reasons for these differences are due to the sampling locations, i.e., the joint center area (42) and the areas outside the TMAZ of the joint.

### 3.7. Temperature Distribution Model

A numerical simulation of the AZ91 friction stir welding was developed based on the model previously published by the authors that was successfully applied to the FSW of other similar and dissimilar material systems [52]. The current simulation follows the same approach for assigning boundary conditions for flow velocities around the tool and for solving for flow stress, viscosity, strain rate, and temperature as the authors’ prior simulation. Material inputs for the simulation, i.e., Sheppard–Wright parameters, Zener–Hollomon parameters, heat capacity (as a function of temperature), thermal conductivity (as a function of temperature), solidus temperature, coefficient of friction, etc., were taken from [22,53,54,55,56,57,58].

**Figure 13:**
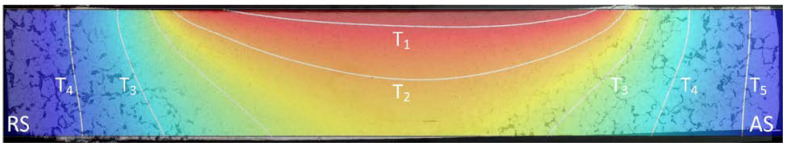
Temperature distribution on cross-section of weld.

**Figure 14:**
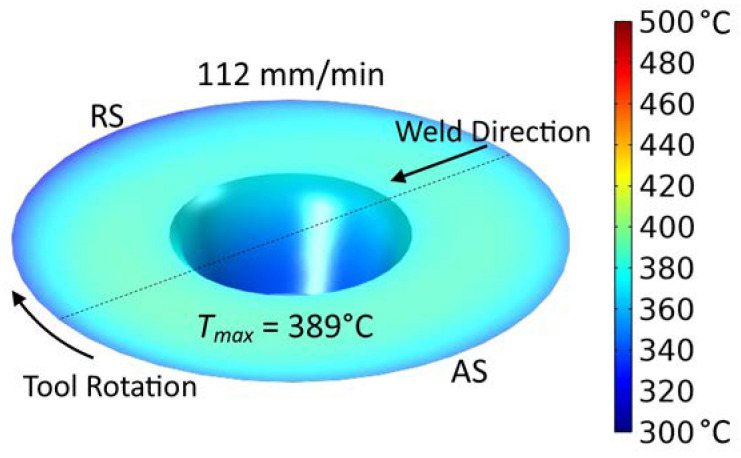
Temperature distribution on FSW tool.

Figure 13 shows a cross-section of the weld. Isotherms and colors represent the temperature distribution applied in this figure, whereas Table 10 presents the values for individual isotherms. As expected, the highest temperature occurs at the weld face. When analyzing the arrangement of the T1 and T2 isotherms, the asymmetric arrangement with respect to the axis of the weld should be pointed out. Higher temperatures occur slightly further from the weld face on the advancing side. In turn, Figure 14 shows the temperature distribution at the tool/workpiece interface taken from the simulation. The highest temperature is approx. 390 °C and is present at the shoulder/workpiece surface. The temperature distribution across the interface results from the flow of plasticized material under the action of the tool. Cooler material ahead at the leading edge of the approaching tool is initially swept to the retreating side. Then, the rotating action of the tool heats the material as it brings it to the trailing edge and then to the advancing side where it is deposited and/or introduced into the weld zone by the pull of the pin. The temperature distribution on the tool/workpiece interface, therefore, is asymmetric with higher temperatures shifted toward the trailing edge and advancing side. 

**Figure 15:**
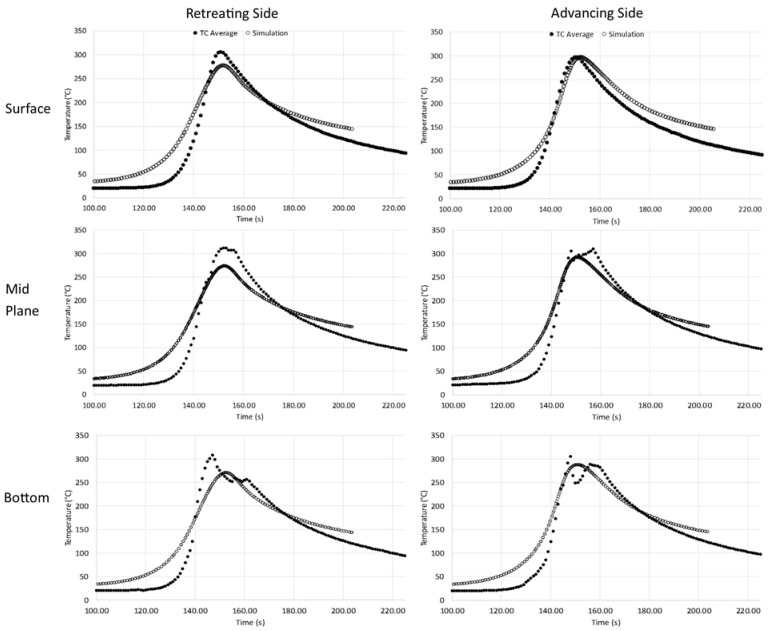
Results of measurement and calculation temperatures of the process.

The temperatures obtained from the model calculations have been compared with the temperature measurements made during welding. Measurements were made using thermocouples inserted into the welded material in various locations enabling the determination of the temperature distribution. Eight thermocouples were used for the measurements—four on the advancing side and four on the retreating side of the joint. Figure 15 displays the curves showing the thermal cycles on both sides of the joint and in relation to the distance to the weld face. The lighter curves are calculated values, while the black curves are measured data. The curve fit is good on both sides of the joint, especially on the advancing side. However, differences may result from the actual location of the thermocouple relative to the planned location. The results indicate that the temperature on the retreating side is lower than that on the advancing side. The cooling rate in the range of 100 °C from the maximum temperature for the presented graphs is approx. 4.5–5 °C/s.

## 4. Discussion

Many studies on the FSW of the AZ91 alloy have been carried out for higher tool rotation speeds, i.e., above 700 rpm. At the same time, the welding linear speeds were relatively low, i.e., several dozen millimeters per minute. References to these studies are described in [39], but also in later works, e.g., [59]. 

This paper describes the effect of a low rotational speed of 355 rpm and relatively high linear speed on welding and properties of an FSW joint. The ratio of rotational speed to linear speed is approx. 3.2 for the tested joint. As described in [59] based on research and numerical modeling for rotational speeds of 750–1000 rpm, the mean welding and transverse forces are inversely proportional to the increase in the rotational speed. This is due to a decrease in the yield strength of the material as more heat is generated during welding. In addition, the amount of material moved and the size of the FSW tool also affect the welding forces and torques. In the present study, the tool dimensions were relatively large—8 mm diameter for the pin, and 24 mm diameter for the shoulder—and a relatively low rotational speed was used. This explains the high values of forces and torques during welding (Figure 3 and Table 6). These observations are confirmed by other tests; e.g., for the AZ91 alloy, an average axial force of approx. 14 kN was observed for welding parameters of 1400 rpm/25 mm/min, using a tool with the following dimensions: pin diameter of 5 mm, pin length of 4.8 mm, and shoulder diameter of 18 mm [60].

**Figure 16:**
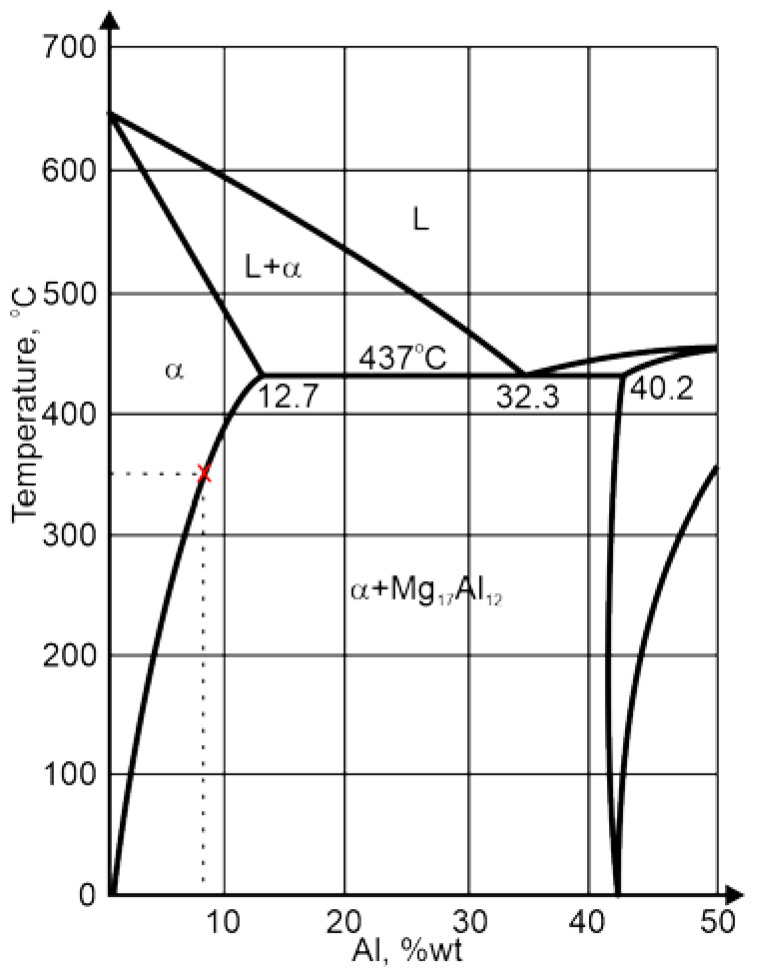
Mg-Al phase equilibrium system.

The study of the joint macrostructure (Figure 4) showed good quality of the weld, i.e., without defects. The structure of the weld is typical for welds made with a tool with a cylindrical pin. In the cross-section, the weld nugget and the thermomechanically affected zone are less visible compared to other test results of FSW welds of Mg alloys: AZ91 [61], AZ31 [62], or with the alloy addition Sn (ATZ511) [63]. However, a significant difference was observed between the areas at the weld face and the rest of the thermomechanically affected zone (TMAZ). The weld nugget has a fine-grained structure, which is observed in microscopic examinations (LM and SEM-EBSD) (Figure 5c, Figure 6b and Figure 7). A characteristic feature is the locations of the Mg_17_Al_12_ phase at the grain boundaries [64]. The occurrence of this phase in the AZ91 alloy and other magnesium alloys with Al addition, e.g., AZ31 and AZ61, is confirmed in many scientific studies [17,22,27]. Moreover, it was also shown that the Mg_17_Al_12_ can occur in three morphological types: lamellar, spherical [29], and eutectic [31]. The higher the Al content in the alloy, the greater the amount of this phase that can precipitate at the grain boundaries [30]. Therefore, the finding in the weld nugget of thin layers of this phase on grain boundaries (in the tested alloy, it is about 9% Al) proves that this area was subject to recrystallization; that thermodynamic conditions were reached, enabling complete dissolution of Al in the Mg matrix; and that rapid cooling then occurred, limiting diffusion and preventing the formation of clusters and large precipitates of the Al-Mg phase. This thesis is also confirmed by the analysis of the temperature distribution in the joint performed using a numerical simulation (Figure 13 and Table 10). The T2 isotherm in this figure is 350 °C. In reference to the Mg-Al equilibrium system (Figure 16), this temperature is on the line of maximum solubility of Al in the Mg matrix for approx. 9% wt. Al, which is the content of this element in the examined AZ91 alloy. In turn, the isotherm T1 is a temperature of 375 °C. The isotherm is located in the area where there are bands of precipitation and large clusters of them (the cross-section (Figure 4)). The reason for the formation of such a microstructure is not only the elevated temperature, but also two other factors related to the influence of the shoulder—the movement of the material and the maintenance of the elevated temperature for a longer time due to the large diameter of the shoulder (24 mm). As shown by the numerical simulation, the surface temperature of the shoulder reaches 389 °C (Figure 13). For the formation of bands and larger precipitates, however, the mechanical impact of the shoulder will be of the greatest importance. This conclusion is drawn from the observation that there are more precipitation bands on the retreating side than on the advancing side, despite the fact that, according to the model, there is a slightly higher temperature near the surface on this side (Figure 13). The fine microstructure also indicates a high degree of plastic deformation in this weld area (Figure 6a compared to Figure 6b). In addition, in this area of the weld, particles containing manganese were observed (Figure 11c,d). According to phase analysis of the AZ91 microstructure described in the literature, these may be particles of the intermetallic phase Al_4_Mn [17]. However, in the AZ31 alloy, the presence of the Al_8_Mn_5_ phase was recognized [65,66].

Similar simulations of the temperature distribution for the AZ91 alloy are presented in [60]. Much higher rotational speeds of the tool (1400 and 710 rpm) and very low linear speeds (25 and 50 mm/min) were analyzed. The results show the dependence of the temperature on the rotational speed, as the temperature reached above 560 °C for the rotational speed of the tool of 1400 rpm and was significantly less (413 °C) for the speed of 710 rpm. This is a value comparable to that presented in Figure 13 and Table 10, but the pin and shoulder diameters of the FSW tool analyzed in work [60] were smaller by 3 and 6 mm, respectively.

The microstructure near the weld surface, mentioned above, will exhibit worse properties than the microstructure of the weld nugget. It is related to the more prominent influence of particles in the cracking process. The mechanism of material cracking from small particles is driven by crack propagation along the material/particle boundary (Figure 11a). Large particles, on the other hand, themselves cracked, showing their brittleness and relatively strong cohesion with the matrix (Figure 11b). Particle banding may in turn result in reduced cohesion or even adhesive cohesion. This can cause delamination under load on the joint. Such a mechanism is suggested by the delaminations observed in the fractures (Figure 10a). The fracture in this critical part of the weld can be described as ductile, although the plasticization of the material is very small because there are no characteristic dimples (craters) resulting from the flow of the material matrix. Although magnesium alloys are not excessively ductile at 20 °C, the microstructure of the AZ91 alloy with greater ductility can be obtained. This is indicated by the results of fracture tests described in [67], in which the types of fractures of the AZ91 layer processed with friction stir processing (FSP) with different rotational speeds of the pin were compared. A similar greater ductility of fractures was also found for the magnesium alloy ME20M [68].

As shown in [60], in this part of the joint, the most significant deformation of the material occurs during the FSW process. A clear asymmetry of the FSW joint is also shown in [69]. On the other hand, in [62,63], the zone near the shoulder is distinguished as a mixing zone or shoulder-affected zone due to significant stirring. Literature analysis shows that this joint area is created in more challenging conditions than other areas. The obtained results indicate, however, that the properties of this part of the joint may be worse. The worse joint properties on the advancing side relate to lower ductility, hardness, and strength. This is confirmed by the results of hardness measurements (Figure 8), according to which the hardness of the advancing side is approx. 60 HV0.1, while the hardness in the weld axis from the retreating side is higher and reaches approx. 80 HV0.1. No correlation is noticed when comparing the hardness distributions with the temperature distribution model (Figure 13). The greater hardness of the material on the retreating side is not associated with a significantly different temperature on this side compared to the advancing side. This means that the differences in hardness are determined by the degree of plastic deformation in individual areas of the weld. The second comparison was made with the results of micromechanical tests (Table 8 and Table 9). There is a correlation with the hardness distribution; i.e., the retreating side shows a strength of up to approx. 300 MPa, i.e., higher by approx. 40 MPa. It is interesting to relate these values to the strength of the parent material tested in the same direction (Parent-L, Table 7). The “macroscopic” strength is 2 times lower than that found in micro-areas. It should be noted, however, that the joint areas were heat cycled, and the central part was also wrought. Another hypothesis that can be considered correlates with the easier crack nucleation (during the tensile test) in a macro specimen than in a small volume of a micromechanical specimen. This is related to the morphology of the microstructure; i.e., the microstructure consists of directionally growing, relatively large dendrites with segregation between them. There may also be possible micro-discontinuities (microshrinkage, interdendritic microshrinkage, or transgranular microshrinkage porosity). A micro specimen contains far fewer of these microstructural elements due to its small volume, and, therefore, there are fewer potential places for crack nucleation. In reference to the results from the literature, it should be noted that the hardness of the AZ91 alloy may range from 52 HV to 84 HV in the as-cast state [18,39,64] and from 85 HV to 105 HV after the FSW process [39,67]. On the other hand, the strength of the alloy after the FSW process may reach 216 MPa in tests with standard-size specimens [39], so the obtained results of 204 MPa for the joint (Table 7) and the average value of data in Table 8 (224 MPa) are comparable.

In comparing the strength of the tested joint with other materials, it should be noted that it is relatively high in the micro-areas of the joint (300 MPa). For example, 365 MPa tensile strength was obtained for the Al/Cu gradient composite [70]. Similar results are found for Al/Cu/SiC laminated composites, whose ultimate tensile strength (UTS) ranges from 150 to 340 MPa [71]. In turn, for a Mg-SiCw/Cu composite fabricated by a combination of casting and severe plastic deformation (SPD), strength was increased to 230 MPa; and with the hardening phase SiCw, strength was increased to 310 MPa [72]. Another Mg alloy, AZ60, was reinforced with particles and exhibited a UTS of 350 MPa [73]. For a layered composite, namely the Mg/Al7075/B4C/Pb composite produced by a combination of coating and severe plastic deformation (SPD) processes, a maximum strength of 170 MPa was obtained [74]. Comparing the obtained result to non-composite metallic materials, 1050 Al alloy shows a UTS of 110 MPa; pure Cu, 179 MPa; and Mg AZ31B alloy, 170 MPa [75].

SEM analyses of specimen fractures supplemented the micromechanical tests (mentioned earlier). Examples of such tests are shown in Figure 12. Individual photos (Figure 12a–c) refer to samples from series 74, 42, and 13. For these series, exemplar strengthening curves are also presented in Figure 9. Fractures of samples from places not subject to plastic deformation (74 and 13 (Figure 2a)) show brittleness typical of the casting microstructure. A detailed SEM analysis shows a slight flow of the material in micro-areas, which is confirmed by the strengthening curves, where the plastic deformation range can be distinguished (Figure 9, curves 74 and 13). On the other hand, at the fracture of the specimen from the middle part of the weld (Figure 12b), there are no large cleavage planes. This shows plasticity consistent for an alloy based on magnesium deformed at room temperature. In this case, this result can also be seen on the strengthening curve (Figure 9, curve 42). Despite the limitations in the plasticity of the AZ91 alloy, the material wrought in the FSW process increases its plasticity 2 or 3 times in relation to the as-cast state of the microstructure (Figure 9). In the case of FSW welds, tests using small sample sizes allowed the strength of individual joint areas to be measured. In studies of Mg alloys, the stretching of small sample sizes was used, for example, to study the anisotropy of the mechanical properties of the AZ91 alloy [29] or damage due to low cycle fatigue of the AZ31 alloy; this study also defined this type of test as a quasi-in situ method [33].

The test results presented above complement the existing knowledge on the use of FSW technology for welding magnesium AZ91 alloys, the properties of such welds, and the testing methods. Obtaining welds at lower rotational speeds and relatively high linear velocities of FSW tools constitutes a new achievement for this material system. The numerical simulation for temperature distribution developed for these parameters correlates well with experimental measurements and provides insight into the microstructural behavior of the weld. Further, detailed micromechanical tests, uncommon in the current literature, were performed, proving their efficacy in the study of elements with complex structures, including FSW joints.

## 5. Conclusions

Based on the performed research, analysis of results, and review of the literature, the following conclusions are drawn:

- It is possible to obtain a good-quality and good-strength FSW joint of the magnesium alloy AZ91 by using a relatively low rotational speed of the tool, a higher linear speed, and a tool with a larger diameter of the shoulder and pin than those indicated in other research results published in the literature.
- For the utilized welding conditions, the temperature during welding reaches values that slightly exceed the maximum solubility temperature of 9 wt% of Al in a magnesium matrix. The re-precipitation of Al and the formation of the intermetallic phase occurs faster near the weld face.
- The hardness is generally lower on the advancing side. The results of hardness measurements are consistent with the results of strength tests obtained in micromechanical tests.
- Micromechanical tests in the field of static tensile tests can be used to test the micro-areas of FSW joints.
- The developed numerical model of the temperature distribution during FSW welding for the tested parameters and the tool matches the experimental results and can be used to analyze the FSW process.
- With the welding conditions used in the experiment, the forces and torque occurring during welding are greater than those reported in the literature.

## Figures and Tables

**Table 1:** AZ91 physical properties [25].

Type	Value	Unit
Density	1.81	Mg/m^3^
Thermal expansion coefficient	27 × 10^−6^	K^−1^
Thermal conductivity	84	Wm^−1^K^−1^
Specific heat	1000	Jkg^−1^K^−1^
Specific resistance	141	nΩm
Young modulus	44 (45) ^1^	GPa
Poisson ratio	0.35 (0.33) ^1^	
Melting point range	470 ÷ 595	°C
Vibration damping	0.2	

^1^ According to [26].

**Table 2:** AZ91 mechanical properties [25].

Type	Value	Unit
No heat treatment		
R_p0.2_	83	MPa
R_m_	117	MPa
Strain	2	%
T4 heat treatment ^1^		
R_p0,2_	125	MPa
R_m_	260	MPa
Strain	9	%
T6 heat treatment ^1^		
R_p0.2_	170	MPa
R_m_	270	MPa
Strain	4.5	%

^1^ T4—400–420 °C, 16–24 h; T6—400–420 °C, 16–24 h cooling in air, 180–210 °C, 8–16 h.

**Table 3:** Chemical composition of AZ91 magnesium alloy, wt%, balance to Mg.

	Al	Zn	Mn
Standard	8.1–9.3	0.4–1.0	0.17–0.35
SEM-EDS ^1^	9.55	0.73	0.95

^1^ Average value of 4 measurements of AZ91 alloy used in the experiments.

**Table 4:** Parameters of welding and quality.

Rate of Rotation (rpm)	Linear Velocity (mm/min)	Defects
355	112	no
355	140	no

Tilt of the tool 1.5°.

**Table 5:** Dimensions (mm) of tensile test specimens relative to Figure 1 and Figure 2.

Type	A	B	C	D	E	F	R	I
Micro	0.7–0.86	0.7–0.86	3.95	3.95	7	6	0.5	-
Parent (L, V)	16	6	24	25	88	70	12.5	-
Macro-V	16	6	24	25	88	70	12.5	3 ^1^
Macro-L	25	6	35	25	63	40	12.5	-

^1^ Radius of the bottom of the notch is 0.32 mm.

**Table 6:** Forces and tool torque of welding—average values.

Weld Force (kN)	Down Force (kN)	Tool Torque (Nm)
2.7	34.1	46.2

**Table 7:** Tensile test results, Rm.

Specimen	Rm (MPa)
Parent-V	164
Parent-L	81
Macro-V ^1^	169
Macro-L	204

^1^ Specimen with notch—Figure 1b, Table 5.

**Table 8:** Tensile strength Rm (MPa) (Micro specimen, Figure 1b) for particular locations in weld presented in Figure 2a; Dff—distance from weld face.

Ret. Side			Center	Adv. Side			Dff (mm)
**201** ± 23.2	**270** ± 4.5	**276** ± 16.2	**294** ± 2.3	**270** ± 22.9	**210** ± 24.1	**168** ± 26.9	1.35
**162** ± 20.7	**235** ± 12.3	**297** ± 4.2	**277** ± 3.6	**272** ± 5.4	**195** ± 5.0	**133** ± 8.8	2.45
**128** ± 27.0	**226** ± 3.0	**298** ± 3.2	**274** ± 1.6	**278** ± 1.7	**188** ± 44.7	**167** ± 13.6	3.55
**146** ± 51.0	**178** ± 29.9	**303** ± 5.1	**295** ± 0.5	**261** ± 5.6	**145** ± 30	**150** ± 8.0	4.65

**Table 9:** Yield point Rp0.2 (MPa) (Micro specimen, Figure 1b) for particular locations in weld presented in Figure 2a; Dff—distance from weld face.

Ret. Side			Center	Adv. Side			Dff (mm)
**89** ± 7.1	**121** ± 5.9	**130** ± 4.4	**140** ± 8.5	**137** ± 10.6	**101** ± 18.9	**66** ± 9.8	1.35
**79** ± 0.2	**98** ± 2.6	**115** ± 0.8	**123** ± 2.5	**123** ± 2.3	**85** ± 4.4	**67** ± 11.9	2.45
**67** ± 11.9	**91** ± 5.7	**116** ± 3.8	**114** ± 2.2	**142** ± 4.1	**84** ± 18.5	**76** ± 8.1	3.55
**77** ± 7.8	**82** ± 7.7	**135** ± 2.5	**105** ± 4.2	**109** ± 3.6	**70** ± 16.1	**76** ± 12.6	4.65

**Table 10:** Temperature for isotherms in Figure 13.

Isotherm	T1	T2	T3	T4	T5
Temperature °C	375	350	325	300	275

## Data Availability

All data are stored at Cracow University of Technology, Poland, and AGH University of Science and Technology, Poland.
